# Immune response after autologous hematopoietic stem cell transplantation in type 1 diabetes mellitus

**DOI:** 10.1186/s13287-017-0542-1

**Published:** 2017-04-18

**Authors:** Lei Ye, Li Li, Bing Wan, Minglan Yang, Jie Hong, Weiqiong Gu, Weiqing Wang, Guang Ning

**Affiliations:** 1 The Department of Endocrinology and Metabolism, Ruijin Hospital, Shanghai Jiao-tong University School of Medicine, Shanghai Institution of Endocrine and Metabolism Diseases, Endocrine and Metabolic E-Institutes of Shanghai Universities and Key Laboratory for Endocrinology and Metabolism of Chinese Health Ministry, No. 197 Ruijin 2nd Road, Shanghai, 200025 People’s Republic of China; 2 The Shanghai Institute of Immunology, Institutes of Medical Sciences, Shanghai Jiao-tong University School of Medicine and Key Laboratory of Stem Cell Biology, Institute of Health Sciences, Shanghai Institutes for Biological Sciences, Chinese Academy of Sciences & SJTUSM, Shanghai, People’s Republic of China; 3 The Chinese Academy of Sciences, Shanghai Jiao Tong University School of Medicine, Shanghai Institutes for Biological Sciences, Laboratory of Endocrinology and Metabolism, Institute of Health Sciences, Shanghai, People’s Republic of China

**Keywords:** Hematopoietic stem cell, Type 1 diabetes mellitus, Immune response, Th1 cell, Th17 cell, Regulartory T cell

## Abstract

**Background:**

This study explored the details of the immune response after autologous hematopoietic stem cell transplantation (AHSCT) treatment in type 1 diabetes mellitus.

**Methods:**

Peripheral blood mononuclear cells (PBMCs) from 18 patients with type 1 diabetes mellitus were taken at baseline and 12 months after AHSCT or insulin-only therapy. The lymphocyte proliferation, mRNA expression and secretion of pro-inflammatory and anti-inflammatory cytokines belonging to T-helper type 1 (Th1), T-helper type 17 (Th17) and regulatory T (Treg) cells in PBMC culture supernatants were assessed.

**Results:**

Compared with patients receiving insulin-only treatment, the patients receiving AHSCT treatment showed better residual C-peptide secretion, lower anti-GAD titers and less exogenous insulin dosages after 12 months of follow-up. AHSCT treatment was associated with significantly reduced Th1 and Th17 cell proportions as well as decreased IFN-γ, IL-2, IL-12p40 and IL-17A levels in the PBMC culture supernatants (all *P* < 0.05). Although there was no significant Treg cell expansion after AHSCT treatment, we observed increased IL-10, TGF-β and Foxp3 mRNA expression and increased TGF-β levels. However, we found no significant changes in the T-cell subpopulations after insulin treatment, except for higher IL-12p40 mRNA expression and a lower proportion of Treg cells.

**Conclusions:**

AHSCT treatment was associated with decreased expansion and function of Th1 and Th17 cells, which may explain the better therapeutic effect of AHSCT compared with the traditional intensive insulin therapy.

**Trial registration:**

Clinicaltrials.gov NCT00807651. Registered 18 December 2008.

## Background

Type 1 diabetes mellitus is an autoimmune disease in which autoreactive immune cells attack pancreatic beta cells, eventually causing complete insulin deficiency [1]. CD4^+^ T cells play a pivotal role in this pathogenesis. Specifically, they predominate in the early phase of insulitis, causing or accelerating disease onset in young NOD mice [2]. CD4^+^ T cells recognize antigenic peptides presented by HLA class II on the surface of antigen-presenting cells, interact directly with these cells via costimulatory and adhesion molecules, produce cytokines and chemokines, and lead to local inflammation [3]. The inflammation damage is compensatory until the amount of the beta cell mass is insufficient to maintain glucose hemostasis, which is followed by overt type 1 diabetes mellitus and severe complications. Current therapies mainly include insulin replacement; however, they have failed to diminish immune damage.

The first case that implemented autologous hematopoietic stem cell transplantation (AHSCT) in a type 1 diabetes mellitus subject was executed by Voltarelli’s group in 2003 [4, 5]. Since then, more studies have been performed to evaluate the therapeutic effect of AHSCTs. These studies consistently demonstrated a well-improved endogenous beta cell function [6–8]. Burt et al. [9] speculated that AHSCT may shift the balance between destructive immunity and tolerance through undefined mechanisms. Brinkman DM et al. [10] showed that AHSCT led to a predominance of tolerating autoreactive T cells and restoration of the CD4^+^CD25^+^ immunoregulatory network in juvenile idiopathic arthritis (JIA) patients. de Oliveira et al. [11] found that AHSCT upregulated fas/fasL and downregulated anti-apoptotic bcl-xL genes expression in peripheral blood mononuclear cells (PBMCs).

Although the clinical and laboratory outcomes are constantly being updated, the exact mechanism of AHSCT is lacking. Here, we explored the immune responses by investigating the change of the peripheral T-cell subsets after AHSCT [6, 12] and traditional insulin injection in patients with type 1 diabetes mellitus.

## Subjects

Between January 2010 and May 2011, 18 subjects with newly diagnosed type 1 diabetes mellitus (age 12–35 years) were enrolled. Type 1 diabetes mellitus was diagnosed according to the 2011 American Diabetes Association (ADA) criteria [13]. Newly diagnosed diabetes was defined as disease duration < 6 months. The protocol for AHSCT was reported in the previous study [6, 12]. Briefly, hematopoietic stem cells were mobilized with cyclophosphamide (2.0 g/m^2^) and granulocyte colony stimulating factor (10 mg/kg/day), and then collected from peripheral blood by leukapheresis and cryopreserved. The cells were injected intravenously after conditioning with cyclophosphamide (200 mg/kg) and rabbit antithymocyte globulin (4.5 mg/kg). Eight patients received AHSCT treatment (AHSCT group) and 10 patients received traditional insulin injection (Insulin-only group). The age, sex ratio, basal daily insulin dose and blood glucose and C-peptide levels were matched in the two groups. Fifteen normal controls with matched age, gender and BMI were enrolled. The board of medical ethics of Shanghai Ruijin Hospital approved the study, and written informed consent was obtained from all of the subjects and/or their parents prior to enrollment.

## Methods

### Biochemical assessment

The daily insulin dose and duration of treatment were monitored and recorded. Serum C-peptide levels were measured by radioimmunoassay using a commercially available kit (Roche Diagnostics, Germany). The serum levels of anti-GAD antibodies were also measured by radioimmunoassay using a commercial kit (RSR Limited, Cardiff, UK), and levels > 7.5 U/ml were considered positive. Hemoglobin A1c was measured by high-pressure liquid chromatography.

### Flow cytometry analysis

First, a portion of the PBMCs was stained with CD3-FITC, CD4-PeCy5, CD8-PE or TCR-α/β-FITC for 30 min in the dark to evaluate the portion of CD3^+^, CD3^+^CD4^+^, CD3^+^CD8^+^ and TCR-α/β lymphocytes. Meanwhile, PBMCs were harvested and incubated with CD4-FITC, CD25-APC or CD127-PE for 30 min on ice to identify regulatory T (Treg) cells. For the intracellular cytokine staining of the T-helper type 1 (Th1) and T-helper type 17 (Th17) cells, PBMCs were stimulated with 50 ng/ml PMA and 500 ng/ml ionomycin (Sigma-Aldrich, St. Louis, MO, USA) for 6 h and then 3 μg/ml Golgi Plug/Brefeldin A (eBioscience, CA, USA) was added to the cells 4 h after stimulation. The cells were incubated with CD3-FITC and CD4-PeCy5 for 30 min at 4 °C in the dark, treated with a fixation/permeabilization solution for 30 min and stained by IFN-γ-APC and IL-17A-PE (all from eBioscience) for 20 min at room temperature [14]. Isotype controls that were labeled with the same fluorescent probe were included for nonspecific binding normalization. Four-color flow cytometry was performed with a Moflo XDP apparatus (Beckman Coulter, Miami, FL, USA), and the data were analyzed with Summit version 5.2.

### Cytokine protein and mRNA measurements

A total of 2 × 10^5^ PBMCs were cultured in 200 μl of complete medium for 72 h after they were stimulated with 5 μg/ml phytohemagglutinin (Sigma-Aldrich). The supernatants were then collected and stored at −80 °C until they were analyzed. The cytokine analyses were run in duplicate with a Procarta® Cytokine Human Multiplex Kit (Panomics, Santa Clara, CA, USA) according to the manufacturer’s protocols. Total RNA was isolated from the PBMCs with the RNeasy mini kit (Qiagen, Hilden, Germany). Quantitative real-time PCR was performed with a Takara SYBR Green PCR Kit. The reaction was run on a LightCycler480 Detection System (Roche, Switzerland), and the data were evaluated with the 2^–△△^CT method. Experiments were performed in triplicate for each data point.

### PBMC proliferation evaluations

Proliferation was evaluated with a water-soluble tetrazolium salt (WST) method by the Cell Counting Kit-8 (CCK-8; Dojindo Laboratories, Kumamoto, Japan) according to the manufacturer’s instructions. PBMCs were resuspended at a density of 2 × 10^6^/ml in a total volume of 100 μl and cultured for 72 h. WST was analyzed by adding the CCK-8 reagent, and the absorbance was measured at 450 nm (OD450) after 3 h with a microplate reader (wavelength 450 nm; Bio-Rad, Hercules, CA, USA). The relative absorbance of the treated group was normalized to the control group. The means ± standard deviation of each triplicate is presented in each graph. All of the experiments were repeated at least three times.

### Statistical analysis

The data are presented as the means (±SD) or medium (95% CI range). The difference between the values amongst the different time points and groups was determined with ANOVAs for normally distributed data or Kruskal–Wallis tests for skewed data. Statistical analysis was performed with the SPSS 17.0 system. *P* < 0.05 for two tails was considered statistical significance.

## Results

### Better islet function in the AHSCT group compared with the Insulin-only group

As shown in Table 1, no significant differences were found at baseline between the patients receiving AHSCT (AHSCT-0 M group) and Insulin-only (Insuin-0 M group) treatments. Twelve months later, patients in the Insulin-only group achieved remarkably decreased HbA1c level (Insulin-12 M vs Insulin-0 M, 7.33 ± 1.42 vs 12.20 ± 3.50%, *P* = 0.002), with no significant changes in the levels of fasting blood glucose, anti-GAD, fasting C peptide, AUCC and insulin dosage (all *P* > 0.05). Compared with the Insulin-only group, patients in the AHSCT group showed remarkably increased fasting C-peptide levels (AHSCT-12 M vs Insulin-12 M, 1.01 ± 0.23 vs 0.60 ± 0.50 nmol/L, *P* = 0.031) and C-peptide AUCs (9.59 ± 2.98 vs 4.76 ± 1.42, *P* = 0.002), and significantly decreased anti-GAD levels (150.4 (31.83–1315.52) vs 1200.0 (725.41–1980.02) units/ml, *P* = 0.043). Although the two groups displayed similar FBG (5.59 ± 1.40 vs 6.04 ± 1.70 mmol/L, *P* > 0.05) and HbA1c levels (6.80 ± 0.60 vs 7.33 ± 1.42%, *P* > 0.05) at baseline, the AHSCT group had less insulin dosages (0.15 ± 0.15 vs 0.52 ± 0.34 U/kg/day, *P* = 0.004) at 12 months.

**Table 1:** Clinical characteristics of type 1 diabetic patients before and after insulin and AHSCT treatments

	Insulin-only group		AHSCT group	
	Insulin-0M	Insulin-12M	AHSCT-0M	AHSCT-12M
Age (years)	20.18 ± 4.02		18.86 ± 1.46	
Gender (female/male)	6/4		5/3	
BMI (kg/cm^2^)	18.28 ± 1.39		19.25 ± 1.11	
FBG (mmol/L)	6.50 ± 2.01	6.04 ± 1.70	6.26 ± 0.67	5.59 ± 1.40
HbA1c (%) (mmol/mol)^a^	12.20 ± 3.50 (109.90 ± 38.42)	7.33 ± 1.42^++^ (56.63 ± 15.45)	11.49 ± 1.46 (102.00 ± 16.06)	6.80 ± 0.60^###^ (51.00 ± 6.73)
Anti-GAD (units/ml)	1230.38 (669.43–1957.29)	1200.00 (725.41–1980.02)	943.68 (294.69–2085.04)	150.4* (31.83–1315.52)
Insulin dose (U/kg/day)	0.66 ± 0.30	0.52 ± 0.34	0.61 ± 0.27	0.15 ± 0.15**^###^
Fasting C-peptide (ng/ml)	0.62 ± 0.25	0.60 ± 0.50	0.71 ± 0.30	1.01 ± 0.23*^#^
AUCC	4.56 ± 2.50	4.76 ± 1.42	5.93 ± 2.54	9.59 ± 2.98**^#^

Data presented as mean ± SD or median (95% CI)

*AHSCT* autologous hematopoietic stem cell transplantation, *0 M* at baseline, *12 M* at 12 months

^#^ *P* < 0.05, ^###^ *P* < 0.001, compared with the AHSCT-0M group

^++^ *P* < 0.01, compared with the Insulin-0M group

*P < 0.05, **P < 0.01, compared with the Insulin-12M group

^a^HbA1c values were calculated as mmol/mol. The NGSP converter is available online (http://www.ngsp.org/convert1.asp)

### Decreased Th1 cell profiles in the AHSCT group compared with the Insulin-only group

The proportions of lymphocytes (40.84 ± 6.93 vs 34.6 ± 4.6%, *P* = 0.019) and monocytes (6.75 ± 1.26 vs 5.50 ± 0.81%, *P* = 0.04) were significantly higher in the type 1 diabetes mellitus patients compared with the normal controls. At baseline, the AHSCT and Insulin-only groups showed similar proportions of WBCs and lymphocytes. After 12 months of treatment, however, the AHSCT group showed lower proportions of WBCs and lymphocytes than the Insulin-only group (4.16 ± 1.17 vs 5.96 ± 1.38%, *P* = 0.011 and 33.64 ± 7.01 vs 42.39 ± 5.90%, *P* = 0.036, respectively). In addition, the AHSCT group showed lower CD3^+^CD4^+^ cell levels (30.59 ± 5.30 vs 43.57 ± 8.70%, *P* = 0.002), but similar CD3^+^CD8^+^ cell (30.83 ± 11.34 vs 27.65 ± 7.93%, *P* > 0.05) and monocyte (7.40 ± 0.32 vs 7.12 ± 1.49%, *P* > 0.05) levels compared with the Insulin-only group. The proportion of TCR(α/β) was significantly decreased in the AHSCT group compared with that in the Insulin-only group after treatment (57.90 ± 10.49 vs 69.94 ± 6.43%, *P* < 0.05) (Table 2).

**Table 2:** Changes in the peripheral blood cell proportion before and after insulin and AHSCT treatments

	Insulin-only group		AHSCT group	
	Insulin-0M	Insulin-12M	AHSCT-0M	AHSCT-12M
WBC (×10^9^/L)	6.01 ± 1.00	5.96 ± 1.38	5.74 ± 1.78	4.16 ± 1.17*
Monocyte (%)	6.37 ± 2.13	7.12 ± 1.49	6.91 ± 1.39	7.40 ± 0.32
Lymphocyte (%)	37.80 ± 8.46	42.39 ± 5.90	42.94 ± 5.55	33.64 ± 7.01*
CD3^+^CD4^+^ T cell (%)	41.96 ± 6.44	43.57 ± 8.70	39.89 ± 8.25	30.59 ± 5.30**
CD3^+^CD8^+^ T cell (%)	31.73 ± 7.99	27.65 ± 7.93	31.49 ± 5.72	30.83 ± 11.34
TCR(α/β) (%)	69.94 ± 6.43	71.95 ± 4.72	72.56 ± 6.46	57.90 ± 10.49*

Data presented as mean ± SD

*AHSCT* autologous hematopoietic stem cell transplantation, *0 M* at baseline, *12 M* at 12 months

*P < 0.05, **P < 0.01, compared with the Insulin-12 M group

**Fig. 1:**
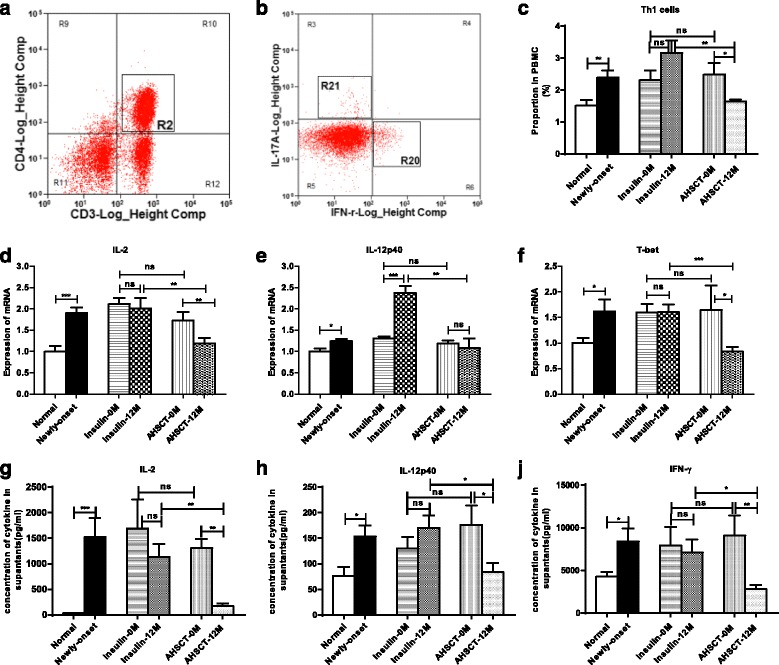
Flow cytometry analysis, cytokine proteins and mRNA measurements of Th1 cells from PBMCs in the AHSCT and Insulin-only groups before and after treatment (*0 M* and *12 M*). **a**, **b** Representative flow cytometry plots of CD3^+^CD4^+^ (R2) and Th1 cells (R20). **c** Proportion of IFN-γ^+^ Th1 cells in PBMCs. **d**, **e**, **f** mRNA expression levels of IL-2, IL-12p40 and T-bet respectively. **g**, **h**, **j** Concentrations of IL-2, IL-12p40 and IFN-γ in the cell culture supernatants respectively. *P < 0.05, **P < 0.01, ***P < 0.001. *AHSCT* autologous hematopoietic stem cell transplantation, *ns* no significance, *PBMC* peripheral blood mononuclear cell

As shown in Fig. 1, R2 represents the CD3^+^CD4^+^ T cells (Fig. 1a) and R20 represents the IFN-r^+^ Th1 cells (Fig. 1b), which were prominently increased in the newly diagnosed patients compared with the normal controls (2.39 ± 0.74 vs 1.52 ± 0.71%, *P* = 0.005; Fig. 1c). The level was significantly reduced after AHSCT treatment (*P* = 0.045); however, no significant changes were found before and after the traditional insulin therapy in the Insulin-only group. At the 12-month follow-up, a much lower proportion of the Th1 subset cells was observed in the AHSCT group compared with the Insulin-only group (1.64 ± 0.14 vs 3.16 ± 0.88%, *P* = 0.005; Fig. 1c).

Additionally, the mRNA level of IL-2/IL-12p40 and the key transcription factor, T-bet, were consistently higher in newly diagnosed T1D patients compared with the normal controls (all *P* < 0.05). They are significantly decreased in the AHSCT group at 12 months compared with the Insulin-only group (IL-2, 1.19 ± 0.37 vs. 2.00 ± 0.74, *P* = 0.009; IL-12p40, 1.08 ± 0.51 vs 2.38 ± 0.36, *P* = 0.002; T-bet, 0.84 ± 0.23 vs. 1.60 ± 0.43, *P* = 0.001, respectively; Fig. 1d–f). The concentrations of IL-2, IL-12p40 and IFN-r in the cell supernatants were all elevated in T1D compared with the healthy group. They were decreased after AHSCT treatment while there was no significant change after insulin therapy in the Insulin-only group. There was a significant decrease in AHSCT-12 M compared with Insulin-12 M (Fig. 1g, h, j).

### Increased Th17 cell proportion was reversed by AHSCT treatment

**Fig. 2:**
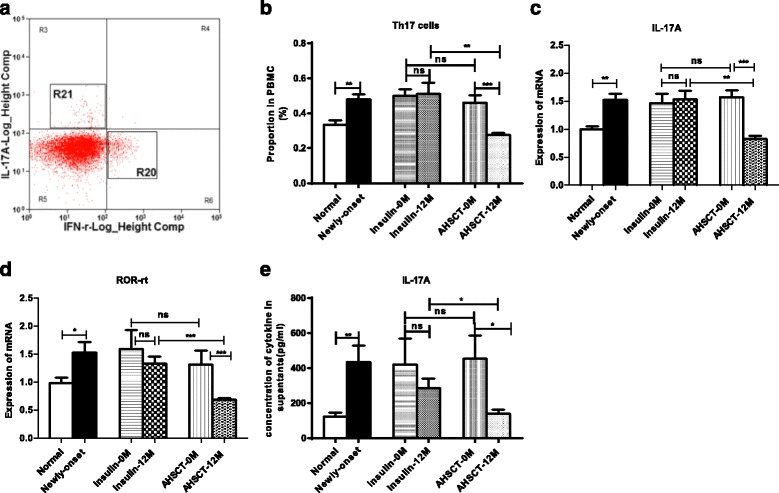
Flow cytometry analysis, cytokine proteins and mRNA measurements of Th17 cells from PBMCs in the AHSCT and Insulin-only groups before and after treatment (*0 M* and *12 M*). **a** Representative flow cytometry plots of Th17 cells (R21). **b** Proportion of CD3^+^CD4^+^IL-17A^+^ Th17 cells in PBMCs. **c**, **d** mRNA expression levels of I IL-17A and ROR-rt respectively. **e** Concentration of IL-17A in the cell culture supernatants. *P < 0.05, **P < 0.01, ***P < 0.001. *AHSCT* autologous hematopoietic stem cell transplantation, *ns* no significance, *PBMC* peripheral blood mononuclear cell

Newly diagnosed T1D patients showed increased Th17 cell proportions compared with the normal controls (0.48 ± 0.08 vs 0.34 ± 0.09%, *P* = 0.001, Fig. 2b). They were significantly decreased after AHSCT treatment (0.28 ± 0.03 vs 0.46 ± 0.09%, *P* = 0.000), while no significant change was found after insulin therapy in the Insulin-only group (*P* > 0.05). As shown in Fig. 2a, R21 represents the plot area for IL-17A^+^ Th17 cells. Importantly, the Th17 cell proportions were much lower in the AHSCT-12 M group than in the Insulin-12 M group (0.28 ± 0.03 vs 0.51 ± 0.15%, *P* = 0.004; Fig. 2b). Furthermore, the mRNA level of IL-17A and transcription factor ROR-rt were downregulated after AHSCT treatment (all *P* < 0.05) and the AHSCT-12 M group was lower than the Insulin-12 M group (IL-17A, 0.83 ± 0.18 vs 1.53 ± 0.36, *P* = 0.002; ROR-rt, 0.7 ± 0.07 vs 1.37 ± 0.28, *P* = 0.000) (Fig. 2c, d).

Significantly higher IL-17A concentrations in the PBMC supernatants was observed in T1D patients compared with the normal controls (435.26 ± 285.44 vs 122.68 ± 75.64 pg/ml, *P* = 0.002). Consistent with the mRNA levels, the IL-17A cytokine levels were significantly reduced in the AHSCT-12 M group compared with the Insulin-12 M group (139.54 ± 63.67 vs 286.90 ± 150.68 pg/ml, *P* = 0.032) and the AHSCT-0 M group (*P* = 0.018) (Fig. 2e).

### Increased Treg cells and higher defined cytokines in the AHSCT group

**Fig. 3:**
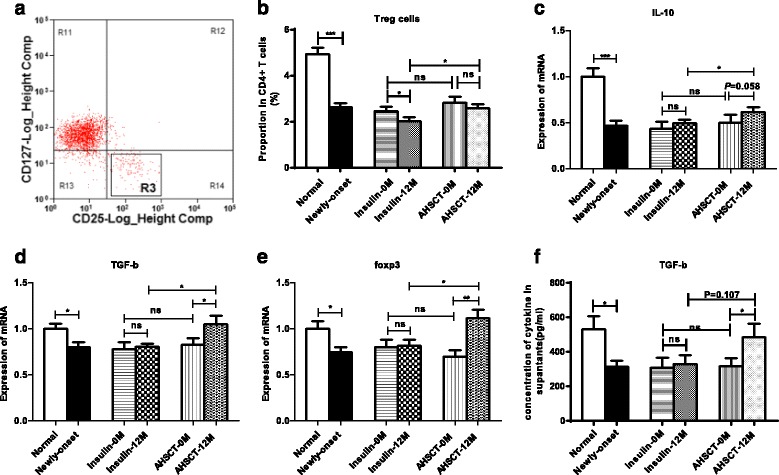
Flow cytometry analysis, cytokine proteins and mRNA measurements of Treg cells from PBMCs in the AHSCT and Insulin-only groups before and after treatment (*0 M* and *12 M*). **a** Representative flow cytometry plots of Treg cells (R3). **b** Proportion of CD4^+^CD25^+^CD127^–^ Treg cells in PBMCs. **c**, **d**, **e** mRNA expression levels of IL-10, TGF-β and foxp3 respectively. **f** Concentration of TGF-β in the cell culture supernatants. *P < 0.05, **P < 0.01, ***P < 0.001. *AHSCT* autologous hematopoietic stem cell transplantation, *ns* no significance, *PBMC* peripheral blood mononuclear cell

As shown in Fig. 3a, R3 represents the plot area of CD25^+^CD127^–^ Treg cells. The proportion of CD4^+^CD25^+^CD127^–^ Treg cells was remarkably lower in the newly diagnosed group compared with the normal controls (*P* = 0.000, Fig. 3b). After treatment, the Treg cells presented a higher proportion in the AHSCT-12 M group compared with the Insulin-12 M group (2.59 ± 0.44 vs 2.02 ± 0.43%, *P* = 0.040). The proportion of Treg cells was decreased after insulin treatment (2.02 ± 0.43 vs 2.46 ± 0.45%, *P* = 0.023) but remained at similar levels after AHSCT treatment (2.59 ± 0.44 vs 2.82 ± 0.64%, *P* > 0.05; Fig. 3b). The IL-10, TGF-β and foxp3 mRNA levels were increased after AHSCT treatment (all *P* < 0.05) and were higher in the AHSCT-12 M group than in the Insulin-12 M group (0.62 ± 0.16 vs 0.49 ± 0.16, *P* = 0.017, 1.05 ± 0.29 vs 0.81 ± 0.11, *P* = 0.033 and 1.12 ± 0.30 vs 0.81 ± 0.21, *P* = 0.016, respectively; Fig. 3c–e).

Finally, we analyzed the IL-10 and TGF-β concentrations in the PBMC supernatants. The TGF-β concentrations of the newly diagnosed patients were much lower than the normal controls (*P* = 0.017, Fig. 3f). After AHSCT treatment, the TGF-β levels were significantly increased (485.33 ± 190.12 vs 317.29 ± 99.09 pg/ml, *P* = 0.036); however, no changes were observed after insulin treatment in the Insulin-only group. A marginally higher level was found in the AHSCT-12 M group compared with the Insulin-12 M group (485.33 ± 190.12 vs 328.87 ± 167.10 pg/ml, *P* = 0.107) (Fig. 3f). The IL-10 levels were undetectable.

### The activated PBMC proliferation levels were downregulated in both groups

**Fig. 4:**
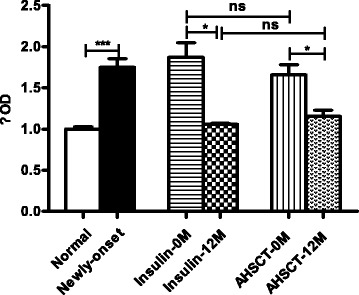
PBMC proliferation levels detected with CCK-8 in the AHSCT and Insulin-only groups before and after treatment (*0 M* and *12 M*). *P < 0.05, ***P < 0.001. *AHSCT* autologous hematopoietic stem cell transplantation, *ns* no significance

As shown in Fig. 4, the proliferation of PBMCs was significantly elevated in the newly diagnosed T1D patients compared with the normal controls (1.75 vs 1, *P* = 0.000). After treatment with either AHSCT or insulin therapy, the proliferation level was significantly downregulated (AHSCT-12 M vs AHSCT-0 M, 1.15 vs 1.66, *P* = 0.012; Insulin-12 M vs Insulin-0 M, 1.06 vs 1.87, *P* = 0.010). However, no significant difference was observed between the two groups after 12 months of treatment (1.15 vs 1.06, *P* > 0.05).

## Discussion

The use of AHSCT to treat severe autoimmune diseases began in the early 1990s [15]. The rationale for AHSCT utilization as a treatment for type 1 diabetes mellitus comes with the double-edged effects of immunosuppressive drugs [16], which are hypothesized to reconstitute immunotolerance and therefore improve insulitis and increase the islet beta cell recovery capacity; however, AHSCT damages the bone marrow, leading to neutropenia and potentially serious infections. In 2008, Burt’s group was the first to apply AHSCT in patients with newly diagnosed type 1 diabetes mellitus [17]. We also previously evaluated the efficacy and safety of AHSCT treatment in type 1 diabetes mellitus [6]. In the present study, we studied the expansion and function of Th1, Th17 and Treg cells after AHSCT treatment.

The CD4^+^ T-cell subsets have divergent CD4^+^ T-cell responses and may contribute to the chronic autoimmune responses in type 1 diabetes mellitus [9, 11, 18]. In our study, although the PBMC distribution was almost the same before treatment, the two groups exhibited an amazing discrepancy 1 year later. The decreased lymphocyte percentages, especially the CD3^+^CD4^+^ T cells, in the AHSCT group dominated the difference. Th17 immunity upregulation has been detected in peripheral blood T cells from children with T1D, and it potentiated both inflammatory and pro-apoptotic responses [19]. Circulating IL-17^+^ β-cell specific autoreactive CD4^+^ T cells were a feature of T1D, and the inhibition of Th17 cells reduced the islet-specific inflammatory T-cell infiltration [20]. In 2011, Wang et al. [21] reported that T-cell vaccination markedly inhibited the retinoic acid-related orphan receptor γt (ROR-γt) mRNA levels as well as the signal transducer and activator of transcription 3 (Stat3) phosphorylation levels, which are the key factors in Th17 cell polarization. At the same time, this treatment decreased blood glucose levels and protected against body weight loss in a diabetic model. Th17 cells may be directly involved in the inflammatory process of the pancreatic islets, causing severe T1D. Th17 cell suppression in the pancreas played a critical role in autoimmune diabetes [21]. Diminished Th17 cell responses underlie multiple sclerosis disease abrogation after HSCT. The post-therapy T-cell repertoire exhibited a significantly diminished capacity for Th17 cell responses, with complete abrogation of new clinical relapses. Additionally, a possible mechanism is the restoration of tolerance to self-antigens, which causes the inhibition of inflammatory cytokine production [22]. With regards to Th1 cells, increased serum CXCL10 concentrations in children with newly diagnosed type 1 diabetes mellitus sign a predominant Th1-driven autoimmune process, suggesting that a Th1 immune response is involved in the initiation of the insulitis [23]; while T-bet-deficient NOD mice were found to have profound defects in diabetogenic CD4^+^ T cells, both in the innate and adaptive immune systems. Moreover, this might lead to the disabilities in initial T-cell priming and proliferation, because T-bet is the critical transcription factor for Th1 polarization in CD4^+^ T cells, which helps to explain the critical role of Th1 cells in insulitis and diabetes development [24]. IFN-γ is produced by Th1 cells, which have also been associated with disease activity in multiple sclerosis (MS). No differences have been observed between T cells from HSCT-treated MS patients and healthy controls in the ability to proliferate and produce IFN-γ after polyclonal stimulation. Meanwhile, signs of immunological disease activity were demonstrated in 10 out of 15 control-treated patients, whereas these changes were not observed in the HSCT-treated group or controls. This lends support to the supposition that HSCT causes the removal of autoreactive T-cell clones [25]. In our study, we observed that Th1 and Th17 cells were significantly increased and activated in the newly diagnosed patients compared with the normal controls. More importantly, we found markedly decreased proportions of these two cell types after AHSCT, which was associated with the downregulated expression of their cytokines (IL-2/IL-12p40/IFN-γ and IL-17A) as well as their transcription factors (T-bet and ROR-rt). These findings suggest a functional role of these two cells in disease progression. Additionally, they had no changes after insulin therapy when blood glucose was controlled, suggesting that the initial high Th1 and Th17 cell-associated cytokines are not related to a specific inflammatory process, which may accompany the metabolic decompensated phase during T1D onset. Because these two cell types mediate extracellular and intracellular pathogen-induced inflammation as well as autoimmunity, we speculate that AHSCT can weaken autoimmune inflammatory reactions and apoptosis, which likely resulted from elimination of the aggressive destruction by the self-recognized effector T cells due to an “immune reset” by stem cells. This effect of AHSCT led to a halt of any further damage to the pancreas and a slowing down of diabetes progression.

We also observed significantly decreased Treg cell proportions in the newly diagnosed T1D patients compared with the normal controls. As reported, Treg cells regulate and ensure immune tolerance in healthy individuals, while Treg cell dysfunction might result in the excessive immune attacks and autoimmune diseases [26]. Deletion of Treg cells could accelerate T1D onset because spontaneous diabetes was exacerbated in both B7-1/B7-2-deficient and CD28-deficient NOD mice [27]. Treg cells can regulate ongoing immune reactions. In NOD mice, the administration of Treg cells could suppress the self-reactive effector T-cell activity and stop the destruction of pancreatic islets, which may potentially benefit T1D [28]. Patients with refractory lupus acquired long-term remission after stem cell transplantation [29]. Their Treg cells returned to the levels observed in the normal subject, accompanied by an almost complete inhibition of the pathogenic T-cell response to major lupus autoantigen from apoptotic cells [29]. In our study, the Treg cell proportion decreased after 1 year of insulin therapy, while it remained stable after the AHSCT therapy. We observed more Treg cells and higher Treg-associated cytokine level in the AHSCT-12 M group than the Insulin-12 M group. Our observations indicated that AHSCT might help Treg cell function and suppress the autoimmune insulitis process, thus leading to a better preservation of β-cell function. However, the absolute amounts of Treg cells in the AHSCT group did not return to normal levels. Cyclophosphamide (CTX) used in the conditioning stage before the stem cell transplantation might impair Treg cells, because Treg cells were more sensitive to CTX than Th cells and CTLs [30]. The administration of purified Treg cells might be a new attempt for immune suppressor therapy [28].

Regarding the PBMC proliferation levels, we found a remarkable reduction after either ASHCT or insulin treatment. These proliferation levels reflected the capacity of the cells to produce clones in vitro. According to Reinhold et al. [31], PBMC proliferation in normal individuals was inversely proportional to the glucose concentration. However, the proliferation indexes were higher in the diabetic patients than in the normal individuals, which could be explained by metabolic defects [32]. Meanwhile, insulin insufficiency may be another reason for elevated PBMC proliferation [33]. Consistent with our finding, this elevation was reversed after metabolic control in both groups [34]. However, there were no significant differences in proliferation between the two groups after 1 year of treatment. This result was unexpected because stem cell transplantation led to decreased proliferation. This discrepancy may be due to the similar metabolic control state, because no significant changes in the blood glucose levels were observed.

There were limitations in this study. The two main limitations of this study included the small number of patients and the duration of the study. A larger sample size and long-term follow-up data are needed in the future. Furthermore, the proliferation capacity of each T-cell subset was not analyzed; the expansion of Treg cells after AHSCT was not consistent with the upregulation of the associated cytokine; and the local pathologic changes in the pancreatic islets were unavailable, which may require further study.

## Conclusions

In summary, our results suggest that AHSCT treatment is tightly associated with the inhibition of T-cell proliferation and pro-inflammatory cytokine production. These changes in the decreased expansion and function of Th1 and Th17 cells have demonstrated the novel immunomodulatory function of AHSCT interactions. When T1D progresses with unavoidable immune damage to the islet cells, AHSCT treatment combined with high-dose immunosuppressive therapy and transplantation of autologous hematopoietic stem cells may play an important role in immune resetting, which could potentially be targeted in therapeutic approaches to T1D. Future clinical studies should shed light on the validity of our hypothesis.

## Abbreviations

AHSCT: Autologous hematopoietic stem cell transplantation
IFN-γ: Interferon gamma
IL: Interleukin
PBMC: Peripheral blood mononuclear cell
TGF-β: Transforming growth factor-β
Th1: T-helper type 1
Th17: T-helper type 17
Treg: Regulatory T
